# Supplementary material for: Preparation of Collagen/Hydroxyapatite Composites Using the Alternate Immersion Method and Evaluation of the Cranial Bone-Forming Capability of Composites Complexed with Acidic Gelatin and b-FGF
Source: Materials (Basel). 2022 Dec 9;15(24):8802. doi: 10.3390/ma15248802 (PMC9787395; doi:10.3390/ma15248802)
Supplement: Supplementary file 1 [file materials-15-08802-s001.zip › materials-2067062-supplementary.pdf]

materials MDPI

Supplementary Material A

Cross-Linking method of Collagen (Col)

Cross-linking of collagen (Col) using physical or chemical methods is frequently performed to improve its durability and strength. Physical crosslinking relies on external high-energy (thermal or radiation) sources; methods include de-hydrothermal treatment (DHT) and ultraviolet or gamma irradiation [14]. Chemical crosslinking involves the formation of intermolecular or intramolecular covalent bonds. The crosslinking reagents are bi-functional organic molecules such as glutaraldehyde (GTA), 1-ethyl-3-[3-dimethylaminopropyl] carbodiimide hydrochloride (EDC) and N-hydroxysuccinimide (NHS), poly-ethylene glycol diglycidyl ether, and genipin [14–16]. Figure A1 illustrates four methods used for Col cross-linking [14], and Table A1 summarizes four methods of type I Col cross-linking based on our experimental results [17] and experience.

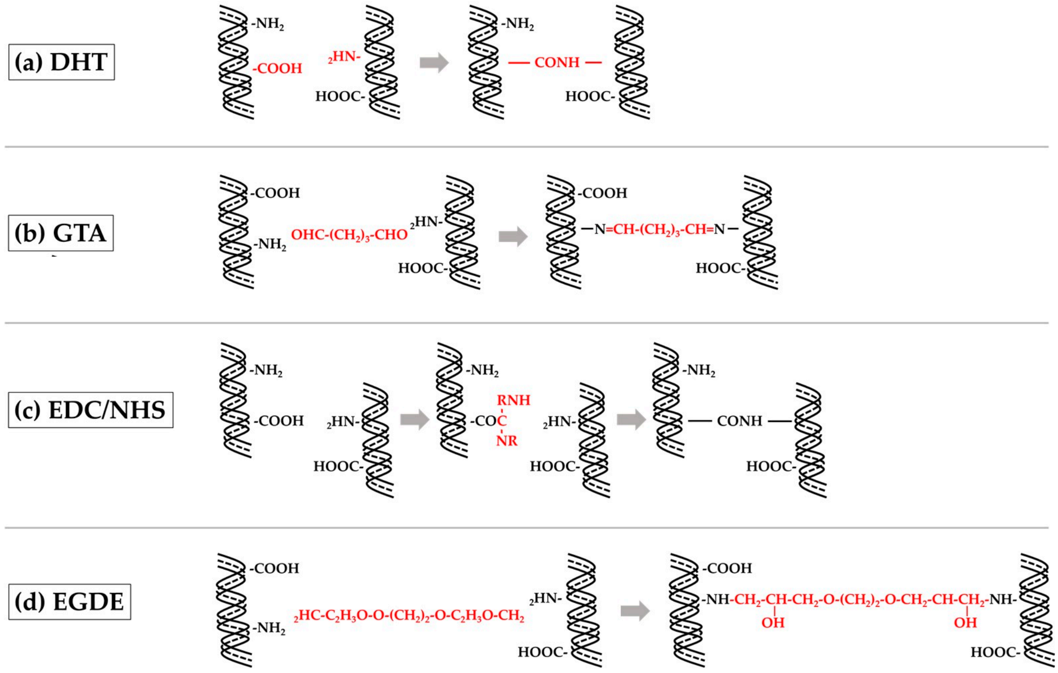

Figure A1. Four cross-linking methods of collagen (Col).

Table A1. Characteristics of four cross-linking methods of collagen (Col).

Cross-linking methods	Toxic concern	Level of cross-linking	Remarks
DHT	Minimum	Small	Routine process
GTA	Large	Large	Better to avoid
EDC/NHS	Minimum	Small	Applicable
EGDE	Small	Medium	Applicable

Note: DHT= de-hydrothermal treatment; GTA= glutaraldehyde; EDC/NHS=1-ethyl-3-[3-dimethylaminopropyl] carbodiimide hydrochloride/N-hydroxysuccinimide; EGDE= Ethylene glycol diglycidyl ether.

Abbreviations

Abbreviations were summarized in the main text.

References

References were written in the main text.

Supplementary Material B

Tissue Engineering and Therapeutic Effects of b-FGF

Three aspects of tissue engineering are illustrated in Figure B1 [28, 29]. Tissue engineering uses a combination of a scaffold, bioactive molecules (i.e., growth factors), and cells to repair, replace, or regenerate lost tissues. Biomaterials (scaffold) are used as a temporary framework that resembles native tissue and provides support for tissue regeneration. A scaffold and growth factors without cells are frequently combined [28–31].

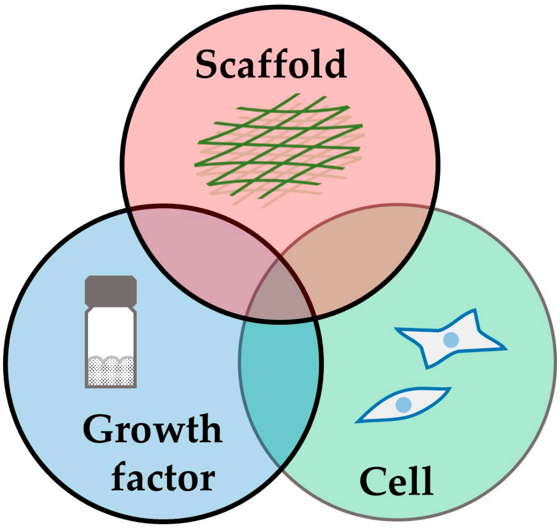

Figure B1. Three elements of tissue engineering.

Figure B2 illustrates the therapeutic effects of basic-fibroblast growth factor (b-FGF), including angiogenesis; accelerated development, growth, and differentiation of mesenchymal stem cells; and wound healing [32–34].

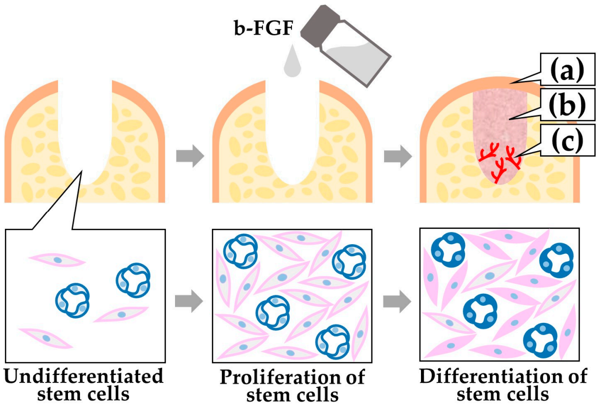

Figure B2. Therapeutic effects of b-FGF.
Note: (a) wound healing promotion, (b) granulation promotion, and (c) angiogenesis promotion

Abbreviations

Abbreviations were summarized in the main text.

References

References were written in the main text.

Supplementary Material C

Self-Preparation of Collagen (Col) Membranes

C1 Introduction

The usage, preparation method and characterization of Col membranes were mentioned here. The formed Col membranes were employed in animal studies. These characterization studies included Col base material (Col control) produced for Col/hydroxyapatite composite granules in the main text.

Figure C1 depicts the methodology of dental bone formation and augmentation. The combined use of Col-based membrane and granules is useful [54–56]. In the present study, we self- produced the membrane and granules from type I Col.

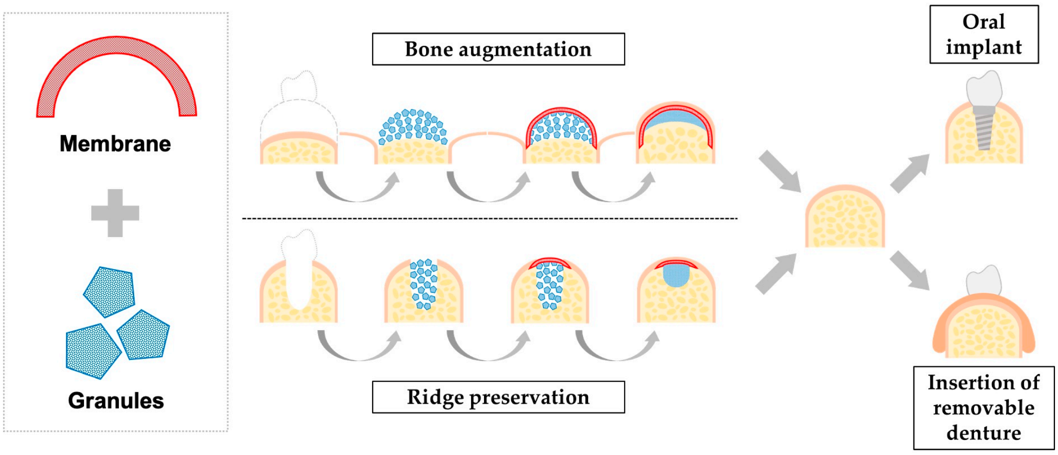

Figure C1. Methodology of bone formation or bone augmentation for dental clinics, using Col.

C2. Materials and methods

C2.1. Materials

For membranes, we used pepsin-solubilized Col derived from porcine skin (Collagen BM; Nitta Gelatin Co., Osaka, Japan), which is a type I atelo-Col/native Col acidic solution (pH = 3, concentration = 5.4 mg/g). Water-soluble ethylene glycol diglycidyl ether (EGDE) (Denacol EX-810; Nagase Chemtex, Osaka, Japan) was used for cross-linking. Chloroform, sodium chloride (NaCl) sodium hydroxide, hydrochloric acid solution and glycine were purchased from Kanto Chemical Co. (Tokyo, Japan).

For granules, we used virus-free medical-grade Col pellets (NMP collagen PS; Nippon Meat Packers Inc., Tokyo, Japan) extracted from porcine skin using pepsin. The pellets mainly comprised type I Col and small quantities of type III Col.

C2.2. Preparation of collagenous membranes

Aqueous type I Col solution (19 mL) was added to a 100-mL freeze-drying container and mixed with chloroform (1 mL) as a porogen. Then, the solution was mixed using a homogenizer (Physcotron; Microtec Co., Chiba, Japan) for 1 min. The solution was poured into three plastic plates (84 × 54 × 12 mm), frozen at −80°C for 12 h, and freeze-dried (FD-5N; Eyela Co., Tokyo, Japan) for 24 h to create membranes. The produced sheet was

defoamed and cross-linked using DHT treatment at 140°C for 6 h in a vacuum dry oven (VO-300; AS ONE, Tokyo, Japan).

The cross-linking solution consisted of distilled water (95 mL), cross-linker (5 mL), and NaCl (11.7g), with the pH maintained at 7 with sodium hydroxide and hydrochloric acid solutions using a pH/ion meter (F-24; Horiba Ltd., Kyoto, Japan). The collagen membrane was mixed with the cross-linking solution (25 mL) in a plastic plate and stored at 4°C for 6, 12, 24, 48, and 72 h for chemical cross-linking. The membranes were quenched by 200 mM glycine buffer solution (25 mL) at 4°C for 60 min to de-activate cross-linking, and washed twice with an ample quantity of distilled water to remove the residual cross-linker. The membranes were repeatedly freeze-dried for cross-linking for 72 h (Col Mmb 72h). Then, the membranes were sterilized using ethylene oxide gas and stored in a desiccator prior to animal experiments.

As a reference material, Col granules (Col control) were produced from medical-grade Col pellets, using 24h DHT method. The detailed preparation process was written in the main text.

C2.3. Characterization of collagenous membranes

C2.3.1. Scanning electron microscopy (SEM) analysis

The outer and cross-sectional surfaces of the membranes, cross-linked for 72 h (Col Mmb 72h) and 24 h DHT medical collagen (Col control), were examined (n = 1 for both) using SEM (SU8010; Hitachi High-Tech Corp., Tokyo, Japan) at an accelerating voltage of 15 kV after plasma-coating with OsO_4.

C2.3.2. Collagenase dissolution tests

The samples (almost 1.5 mg) for membrane experiments cross-linked for 6–72 and 24 h DHT medical collagen (Col control) were dissolved in 0.01 wt% collagenase (S-1; Nitta Gelatin Co.) solution diluted in distilled water (1 mL). The solution was stored in a 1.5-mL microtube in a constant temperature bath at 37°C. The dissolution conditions were visually inspected, and the time to complete disappearance (min) was recorded. The dissolution tests were repeated six times for each sample (n = 6).

C2.3.3. Thermal gravimetry (TG)/differential thermal analysis (DTA)

Thermogravimetry (TG)/differential thermal analysis (DTA) mode thermal analyses were performed on 6 mg of membranes cross-linked for 6–72 or 24 h, including Col Mmb 72h and 24h DHT medical collagen (Col control), using specialized equipment (TG/DTA 6300; Hitachi High-Tech Corp.). The experimental conditions were as follows: atmospheric gas, nitrogen; gas flow rate, 200 mL/min; temperature range, room temperature to 1,000°C; heating rate, 10°C/min; sample holder, open platinum crucible; reference, alumina powder (6.75 mg). The differential thermal gravimetry (DTG) curve was obtained through derivation of TG measurements. Thermal analysis was conducted using a single specimen from each sample (n = 1).

C3. Results

C3.1. Characterization Studies

C3.1.1.SEM

Figure C2 (a) and (b) shows SEM photomicrographs of outer and cross-sectional surfaces of the collagen membrane, chemically cross-linked for 72 h (Col Mmb 72h) and 24 h DHT medical collagen (Col control) used for granule preparation, respectively. The outer surface of the collagen membrane exhibited inter-connected porous structures with mean short and long diameters of 118 and 291 μm (n = 10), respectively [Figure C2 (a)]. The cross-sectional view showed a porous layered structure with short and long diameters of 69 and 288 μm (n = 10), respectively. Other prepared membranes cross-linked for 6–48 h had a similar structure to the membrane cross-linked for 74 h. The outer surface of the medical collagen (Col control) also exhibited porous structures with mean short and long diameters of 27 and 63 μm (n = 10), respectively [Figure C2 (c)]. The cross-sectional view showed a porous layered structure with short and long diameters of 55 and 212 μm (n = 10), respectively [Figure C2 (d)]. The morphology of the self-prepared collagen membrane and Col control was similar to that of the guided tissue regeneration (GTR)/guided bone regeneration (GBR) membranes, as shown in previous studies [57].

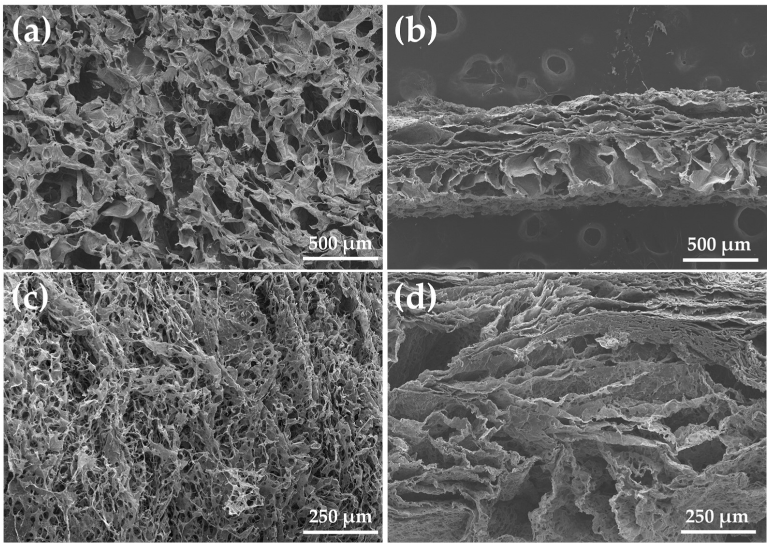

Figure C2. SEM photomicrographs of (a) outer and (b) cross-sectional surfaces of the collagen membrane chemically cross-linked for 72 h (Col Mmb 72 h) (50× magnification); (c) outer and (d) cross-sectional surfaces of 24 h DHT medical collagen (Col control) (100× magnification).

C3.1.2. Collagenase dissolution tests

Figure C3 (a) and (b) shows the collagenase dissolution time of the self-cross-linked membranes with different cross-linking times, and 24 h DHT medical collagen (Col control); and commercial artificial dermis and GTR/GBR membranes, respectively. Data for the latter have been reported previously [57]. Increased cross-linking of the membranes was associated with an increase in the collagenase dissolution time [Figure C3 (a)]. The collagenase dissolution time of artificial dermis was significantly shorter than that of the GTR/GMR membranes [Figure C3 (b)], correlating with *in vivo* dissolution times of 1–2 weeks [49] and 2–6 months [58], respectively. The collagenase dissolution time of the self-prepared Col cross-linked for 72 h and DHT medical Col were comparable to and slightly shorter than that of GTR/GBM membranes, respectively.

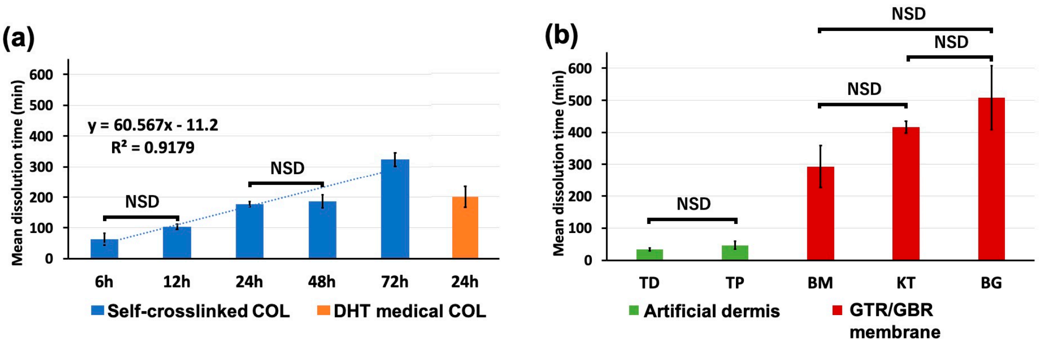

Figure C3. (a) Collagenase dissolution time of self-cross-linked membranes for five different cross-linking times (6, 12, 24, 48, and 72 h) and 24 h DHT medical Col (Col control). (b) Collagenase dissolution time of commercial artificial dermis and guided tissue regeneration (GTR)/guided bone regeneration (GBR) membrane materials. NSD: Not statistically different ($p > 0.05$)
Note: TD = Terudermis (Olympus Terumo Biomaterials Corp., Tokyo, Japan); TP = Teruplug (Olympus Terumo Biomaterials Corp.); BM = BioMend (Zimmer Biomet Dental GK, Warsaw, IN, USA); KT = Koken Tissue Guide (Koken Co., Ltd., Tokyo, Japan); BG = Bio-Gide (Geistlich Pharma AG, Wolhusen, Switzerland).

C3.1.3. TG/DTA

Figure C4 (a) and (b) shows the TG/DTA curves of membrane cross-linked for 72 h (Col Mmb 72h) and 24 h DHT medical collagen (Col control), respectively. Both curves showed DSC endothermic peaks (*1 and *2) at 70°C due to water evaporation and large DTG peaks at almost 320°C (#1 and #2) due to heat decomposition of the cross-linked Col at 200–800°C [59, 60]. A small peak is indicated by "$" in Figure C4 (b). This peak was attributed to a change from the triple helix in Col fibrils to a single random coil [60, 61]. The TG/DTA peaks of the two materials were similar to those of commercial GTR/GBR membranes [57]. Conversely, the TG/DTA peaks of the self-produced membranes cross-linked for 6–48 h were similar to those of commercial artificial dermis [57], which had large DTG and DTA endothermic peaks at 220°C. The specific DTG peak temperature [57] of the self-cross-linked membrane was influenced by the cross-linking time. Longer cross-linking time was associated with a higher DTG specific peak temperature [Figure C4 (a)].

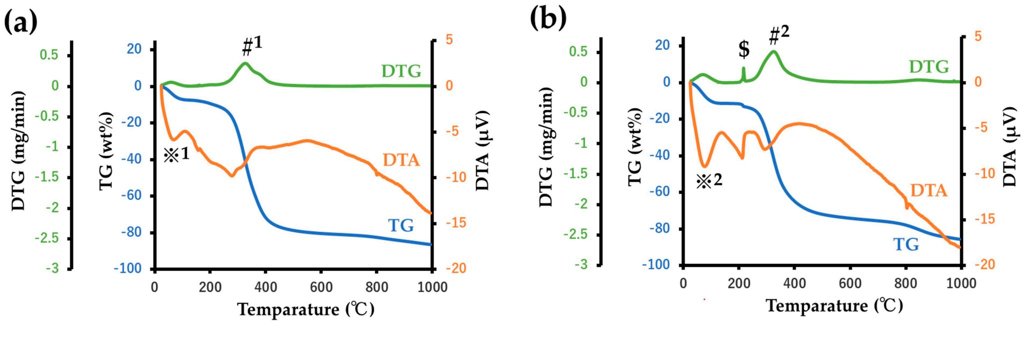

Figure C4. TG/DTA curves of (a) a membrane cross-linked for 72 h (Col Mmb 72h) and (b) 24 h DHT medical collagen (Col control). Note: *1 and *2 indicate endothermic peaks at almost 70°C. # 1 and #2 indicate DTG peaks at almost 320 °C. $ indicates a small DTG peak accompanied with weight loss. DTG curve was obtained through derivation of TG measurements.

Cross-linking for 6, 12, 24, 48, and 72 h was associated with DTG specific peak temperatures of 172°C, 241°C, 241°C, 274°C, and 325°C, respectively. The DTG specific peak temperature of 24h DHT medical Col (Col control) was 316°C. A previous study reported the

specific peak temperatures of two artificial skins (228°C and 301°C) and three GTR/GBR membranes (316°C, 327°C, and 329°C) [57]. The membrane cross-linked for 72 h (Col 72 Mmb 72h) and 24h DHT medical Col (Col control) had similar thermal durability, among other properties, to the GTR/GBR membranes.

C4. Discussion

We developed a bone substitute system consisting of membrane and granules (Figure C1) using animal-derived collagen [62–65] and AG [66]. We did not use commercial Col products to develop the collagenous biomaterials.

For the self-prepared Col membranes, we performed cross-linking for 72 h (Col Mmb 72h). We used chloroform as a porogen to provide the membrane with inter-connected porosities [67] [Figure C2 (a) and (b)]. The cross-linker (EGDE) used was safer than GTA [68], and a safe cross-linker DHT was also used (Appendix A1). The collagenase dissolution time of 24h DHT Col control was comparable to that of GTR/GBR membranes, which are commonly used for > 2 months. In our animal experiments, the membrane chemically cross-linked for 72 h (Col Mmb 72h) disappeared within 8 weeks of implantation in rat cranial bone defects. This was because wound healing with bleeding and clotting, and the formation of granulation tissues, accelerated the bioabsorption of collagen membranes [69, 70]. In the absence of a healing process, the membrane may remain *in vivo* for longer than 2 months, as shown by our preliminary experiments [57]. The use of collagen membrane is important in animal studies because it performs various functions, including absorption of blood, control of intraoperative bleeding, prevention of infection, and wound healing [71]. Additionally, it prevents the dropout of granules placed in rat cranial bone defects. The membrane promotes bone formation in cranial bone defects filled with granules by acting as indispensable auxiliary material. It serves as a protective membrane and disappears within 2–3 weeks to allow healing without hindrance from the remaining material. To develop the collagenous membranes, material characterization [57,72] was used for material design and feedback analysis was used to evaluate the clinical performance. The collagenase dissolution test (Figure C3) and TG/DTA thermal analyses (Figure C4) are useful for measuring the collagenase dissolution time and DTG specific peak temperature, respectively. These measurements indicate *in vivo* longevity under stable conditions and a degree of cross-linking of collagenous materials [73], respectively, which are important for the development of therapeutic collagenous bio-materials.

C5. Conclusion

We self-prepared collagen membranes using chemical cross-linking, to cover bone defects with and without Col-based constructs. Self-prepared Col membrane provided a protective barrier and covering material for the defects.

Abbreviations

Abbreviations were summarized in the main text.

References

References were written in the main text.
